# A retrospective study of choke (oesophageal obstruction) in 64 one‐hump Dromedary camels (*Camelus dromedarius*) in Saudi Arabia

**DOI:** 10.1002/vro2.53

**Published:** 2022-12-25

**Authors:** Mohamed K. Zabady, Turke Shawaf

**Affiliations:** ^1^ Department of Clinical Sciences College of Veterinary Medicine King Faisal University Al‐Ahsa Saudi Arabia; ^2^ Department of Surgery Faculty of Veterinary Medicine Cairo University Giza Egypt

## Abstract

**Background:**

Choke (oesophageal obstruction) is an important oesophageal disorder in large domestic animals. Published studies on choke in the dromedary camel (*Camelus dromedarius*) are few in number and deal with small number of cases.

**Methods:**

Sixty‐four camels with choke were presented to the Veterinary Teaching Hospital, King Faisal University. History, breed, age, sex, duration of obstruction and clinical signs were recorded. The diagnosis was established using examination with a stomach tube, oesophageal radiography and endoscopy. Choke was managed either by using alligator forceps guided endoscope or by cervical oesophagotomy.

**Results:**

Choke was recorded in camels less than 1 year old (84.38%) more than camels more than 1 year of age (15.62%) and complete obstruction more than partial. Most cases had obstruction involving the cervical oesophagus (96.87%). In the majority of obstructive masses, there were pieces of fabric (48.44%) and plastic bags (35.94%). Most obstructive masses were slightly radio‐opaque (62.5%). Surgical and non‐surgical managements were effective (91.3% and 94.44%, respectively) in resolving the choke.

**Conclusions:**

Choke was most likely in animals less than 1 year of age with complete obstruction of the cervical oesophagus. Surgical and non‐surgical methods were effective in resolving the choke in the dromedary camels. It was not practical to use forceps extraction in all adult camels due to the limited length of the alligator forceps.

## INTRODUCTION

Oesophageal obstruction has been described in cattle,^1^ horse,^2^ dromedary camel calf^3^ and water buffalo.^4^ It has also been described occasionally in sheep and goats.^5^ In cattle, food items such as potato, beet root, radish, corn cobs and watermelon rind, without sufficient mastication, have been associated with oesophageal obstruction.^6^ In horses, choke may be a sequel to intraluminal impaction with food material or ingesta.^7^, ^8^ Choke has been described in South American camelids.^9^, ^10^ In alpaca and llama crias, vascular ring anomalies may present with clinical signs similar to those seen with choke^9^; similarly with megaoesophagus in llamas.^10^ In dromedary camel calves, endoscopy has been reported as an effective method for dealing with oesophageal obstruction.^11^ There are limited numbers of published reports and case description of dromedary camels with choke^12^, ^13^, ^14^, ^15^; including two small case series in calves from the authors’ institution.^3^, ^11^

The aim of this study was to describe choke in dromedary camels, including some observations on diagnostic methods, the nature of the obstructive masses; with some comments on the methods of management.

## MATERIALS AND METHODS

### Animals

Sixty‐four dromedary camels (*Camelus dromedarius*) with a diagnosis of choke were presented to the Veterinary Teaching Hospital of the College of Veterinary Medicine‐King Faisal University from 2009 to 2018. The history in all cases included regurgitation of food and/or milk. The breed (Majaheem, Magateer and Omani), age, sex and duration of clinical signs were recorded. The age ranged from 10 days to 8 years; most cases were under 1 year old; consequently, age was designated as one of two groups (less than 1 year and more than 1 year old).

The type of oesophageal obstruction was designated as either complete or incomplete (partial) based on the history, oesophageal radiography and endoscopy. With complete obstruction, the history usually included sudden onset of dysphagia, regurgitation of food, milk and fluids, salivation and restlessness. With partial obstruction, there was regurgitation only of coarse and hard food. The duration of clinical signs varied between 2–4 days (median 3 days) with complete obstruction and 1–5 months (median 3 months) with partial obstruction. The clinical examination included passing a flexible stomach tube in all cases.

### Radiographic examination

Plain oesophageal radiography was performed for all cases. An oesophagogram was carried out in 30 camels using 200–300 ml and 500–600 ml barium sulphate solution (72%, w/v, with water) in young and adult camels, respectively. The solution was administered orally with a dose syringe or by stomach tube.

### Endoscopic examination

Endoscopy was conducted in all camels under 1 year of age (*N* = 54) using a flexible endoscope (VetVu, a unit of Swiss Precision Products), 8 mm diameter, 110 cm long, supported with an inflation and irrigation system and light source. The camel was secured in sternal recumbence and sedated with xylazine 0.2 mg/kg intravenously (I/V) (Seton 2%; Laboratorios Calier, S.A.C./Barcelonés, Barcelona‐Espana). The oral cavity of camel was kept open with a Gunther's mouth Gag (Eickeymeyer, Germany). The endoscope was inserted in the oral cavity, pharynx and oesophagus. Oesophageal fluid was aspirated with a surgical suction unit (New Askir, Italy). The oesophageal mucosa was examined and the lumen was inspected for the nature of obstruction. The obstructive area was washed through the channel of the endoscope using 50 ml NaCl 0.9% solution to visualise the foreign body.

### Non‐surgical management (*n* = 18)

After completion of the endoscopic examination, grasping alligator forceps (Eickeymeyer) with 80 cm long arms were inserted into the oral cavity and oesophagus to grasp the end of obstructive mass to attempt to remove it (Figure S1). The procedure was repeated up to five to six times depending on the type, size and location of obstructive mass. After removal of the obstructive mass, the oesophagus was flushed with 300 ml of saline to ensure patency of the oesophagus.

After the removal of the obstruction, camels received penicillin and streptomycin (Pen & Strep, Norbrook Laboratories, UK) intramuscularly (I/M) as 8 mg/kg procaine penicillin with 10 mg/kg dihydrostreptomycin sulphate, equivalent to 1 ml per 25 kg bodyweight, for 5 days; together with flunixin meglumine (Finadyne solution, MSD Animal Health, North Ireland) I/V, single daily dose of 1.1 mg/kg for 3 days. Camel calves were given access to suckle milk after 12 h.

### Surgical management (*n* = 46)

Cervical oesophagotomy was carried out in 36 camels under 1 year of age that had tightly impacted obstructive masses that could not resolved with endoscopy and in 10 camels over 1 year of age. The anaesthetic regimen comprised sedation with xylazine 0.2 mg/kg I/V, followed by general anaesthesia with ketamine hydrochloride 10% (Ketamidor 10%, Richter Pharma AG, Austria) 2 mg/kg I/V. Each camel was placed on its right side in lateral recumbence and a stomach tube was introduced close to the obstruction. The distal third of the neck was prepared for surgery. A single incision (10 cm long) was made at the ventrolateral aspect at skin and fascia; then, the sternocephalicus and sternothyrohyoideus muscles were bluntly separated while preserving the jugular vein beneath the sternocephalicus muscle. The oesophagus was located dorsolateral to the trachea and under the jugular vein; it was held with two stay stitches. A longitudinal incision (5 cm long) was made in the dorsolateral aspect of the oesophagus through the outer fibrous and muscular layers, then through the inner submucosal and mucosal layers, to expose the lumen. Rochester‐Carmalt forceps were used to extract the obstructing foreign object (Figure S2a,b). The stomach tube was pushed towards the chest to check the patency of the oesophagus and the surgical field was flushed using sterile normal saline solution. The oesophageal mucosa and submucosa were sutured using simple interrupted pattern (in out–out in) using 1–0 polygalactine 910 (Surgicryl, Smi AG, Hünningen 37 Belgium). The outer muscular and fibrous layers were opposed with a simple continuous pattern. Benzyl penicillin powder was dusted into the depth of the wound. The fascia over the muscles was opposed using 1–0 polygalactine 910 in a simple continuous suture pattern. The skin was closed using two silk (Lukens Medical, USA) in an interrupted pattern.

### Postoperative care

Food was withheld for 3 days and maintenance fluid therapy (Lactate Ringer's solution) was infused I/V at a rate of 70 ml/kg/24 h. After 3 days, a soft diet such as milk was advised. Camels received oxytetracycline (Tetracyn, Laboratorios Argos, Santa Fe, Argentina) I/M, 20 mg/kg, every 72 h and flunixin meglumine I/V, once daily, 1.1 mg/kg, for 7 days. The skin sutures were removed 14 days postoperation. The cases were followed up for 6 months via telephone.

## RESULTS

Choke was more common in the Majaheem breed (67.19%; *N* = 43) than the Magateer and Omani breeds and in camels less than 1 year of age (84.38%; *N* = 54) than camels more than 1 year (15.62%; *N* = 10) (see Table 1). The most common type of obstruction was complete (84.38%; *N* = 54).

**TABLE 1:** The number and percentage of breed, sex, age, type of obstruction, site of obstruction and nature of obstructive mass in 64 dromedary camels with oesophageal obstruction

Items	Variable	No.	%
Breed	Majaheem	43	67.19
	Magateer	11	17.19
	Omani	10	15.62
Sex	Male	26	40.62
	Female	38	59.38
Age	<1 year	54	84.38
	>1 year	10	15.62
Type of obstruction	Complete	54	84.38
	Partial	10	15.62
Site of obstruction	Cervical	62	96.87
	Thoracic	2	3.13
Nature of obstructive mass	Pieces of fabric	31	48.44
	Plastic bags and ropes	23	35.94
	Phytobezoar	6	9.38
	Trichophytobezoar	2	3.12
	Rubber material	2	3.12
Radio‐opacity of obstructive mass	Radio‐opaque	8	12.5
	Slightly radio‐opaque	40	62.5
	Radiolucent	16	25
**Management**			
Surgical (*n* = 46)	Resolved cases	42	91.3
	Complicated cases	4	8.7
Non‐surgical (*n* = 18)	Resolved cases	17	94.44
	Complicated cases	1	5.56

*Note*: Including radiographic findings and case outcome.

The flexible stomach tube was valuable in determining the site of obstruction in all cases. Attempts to push the obstructive mass with the stomach tube always failed because it only moved the mass several centimetres and then the mass was stuck in the oesophageal lumen.

Oesophageal radiography revealed the site and the radio‐opacity of the obstructive mass in all cases examined. Choke in the cervical oesophagus was more common that the thoracic oesophagus (96.87%; *N* = 62; 3.13%; *N* = 2, respectively). In terms of radio‐opacity, the obstructive masses were radio‐opaque, slightly opaque or radiolucent. Some obstructive masses were radio‐opaque (12.5%; *N* = 8) such as phytobezoars (Figure S3a), tightly impacted clothing fabric and a piece of rubber. The majority of obstructive masses were slightly radio‐opaque (62.5%; *N* = 40) including pieces of fabric (Figure S3b), trichophytobezoar (compacted hair and plant/food material) and some phytobezoars (compacted plant/food material). Some masses were radiolucent (25%; *N* = 16) (Figure S4a).

In all 30 cases, contrast oesophagram revealed that the barium solution was retained at the site of obstruction in complete oesophageal obstruction, while in partial obstruction, incomplete filling of oesophageal lumen was observed (Figure S4b). Megaoesophagus was detected in one calf at the third intercostal space due to obstruction of the thoracic part of the oesophagus. Endoscopy of camel calves assisted in recognising the nature of obstructive mass and assessing the integrity of oesophageal mucosa.

Non‐surgical management using the alligator forceps guided endoscope was successful in 17 camels under 1 year. These camels had obstructive masses that were not firmly impacted in the lumen of the oesophagus and were within the range of the forceps. Inflammation of the oesophageal mucosa was observed in seven cases. One camel calf had an oesophageal tear after removal of an obstructive mass and the owner advised to slaughter the animal. The tear was associated with an attempt to retrieve an impacted trichophytobezoar.

Surgical management (cervical oesophagotomy) was effective in resolving choke of adult camels and calves with tightly impacted foreign bodies located beyond the length of the forceps (*N* = 42; 91.30%). One adult camel had choke in the thoracic part of the oesophagus; this was resolved by oesophagotomy at the distal oesophagus and introduced long forceps and endoscope from the oesophagotomy incision. A further three camels developed complications with oesophageal fistula in the second week postoperatively at the oesophagotomy site. Food material discharged from the oesophagotomy incision with phlegmon to the surrounding tissues.

The obstructive masses are listed in Table 1 (see Figure S5).

## DISCUSSION

The primary goal of treatment for choke is to relieve the obstruction with minimal trauma to the oesophagus and to limit other complications. In keeping with previous reports, the majority of the oesophageal obstructions in our study occurred in camel calves.^3^, ^11^ This may be due to camel calves ingesting inappropriate materials without adequate mastication. Some greedy animals may bolt a mouthful of feed and try to swallow it without chewing.^16^ The signs of oesophageal obstruction recorded were comparable to those reported in camel calves,^3^, ^11^ adult camels,^12^, ^14^, ^15^, ^17^ llama,^16^ horses,^7^, ^18^, ^19^, ^20^ cattle^1^, ^6^ and small ruminants.^21^, ^22^

Both complete and partial oesophageal obstructions were recorded in this study, similar to ruminants^23^ and llamas.^16^ One study only reported complete oesophageal obstruction in camel calves.^3^ Complete obstruction may be due to spasm induced around the obstructing mass.^24^

In this study, the cervical part of the oesophagus was the most common site of obstruction, especially its distal third, cranial to the thoracic inlet. In a previous report on camel calves,^3^ the most common site was caudal to the thoracic inlet. Factors that may contribute to the location of the obstruction may include the length of the cervical oesophagus, which is nearly twice that of the thoracic part. Moreover, oesophageal diameter in the cervical portion is narrow (2.5 cm) compared with thoracic portion (3.9 cm).^25^ Furthermore, camels have a long vertical neck except at its distal third, where it becomes horizontal to enter the chest; such a curvature may increase the possibility of obstruction.^3^

Oesophageal radiography revealed that some obstructive masses were radio‐opaque or slightly opaque and could be detected easily with plain radiography. Barium oesophagram was useful for viewing radiolucent obstructive masses and evaluating the oesophageal condition. This is similar to horses where survey radiography of feed impaction or lodgment of metallic foreign bodies can be a valuable diagnostic method.^20^ Plain and contrast oesophageal radiography in horses^19^ and cattle^6^ can help to diagnose various oesophageal disorders.

Endoscopic removal of oesophageal foreign bodies in the camel calves was successful; similar to previous reports in Holstein bovine calves^26^ and camel calves.^11^ In adult camels, endoscopic removal of oesophageal foreign bodies could not be performed because the length of alligator forceps was 80 cm and the oesophageal length is approximately 150 cm in adult camels.^17^

Cervical oesophagotomy via a ventrolateral approach provides access to the middle and distal cervical oesophagus and proved to be a convenient way for extraction of tightly impacted oesophageal foreign bodies. The cervical part of the camel oesophagus was most accessible to surgery given that it makes up over 60% of the total length of the oesophagus. In horses, the cervical oesophagus constitutes over 50% of the oesophageal length and a ventrolateral approach is more desirable than a ventral approach due to the heavily developed ventral cervical musculature.^20^ Oesophagotomy has been described in camel calves,^3^ horses^20^ and goats.^22^ Three camels developed an oesophageal fistula because of rapid access to the food on the second day postoperatively, leading to food accumulation, infection at oesophagotomy incision and subsequent fistula formation. In horses, oesophageal fistula may result from healing of the oesophagotomy incision.^27^

The obstructive masses were similar to those reported in previous studies of camels.^3^, ^12^, ^13^ It has been reported that potatoes and root crops occasionally cause choke in camels.^3^, ^28^ One case was previously reported due to an injured and paralysed soft palate.^29^ Unlike in dromedary camels, the oesophagus was frequently obstructed in cattle and horses by various feed items including apples, carrots, corn cobs and fibrous feed.^6^, ^23^ Phytobezoars may cause choke in horses^30^ and goats.^22^ This study has helped to extend the findings of previous studies on choke in dromedary camels. It also complements other studies of oesophageal disorders in dromedary camels which have, separately, reported that lesions of sarcocystosis are commonly found in oesophageal tissues.^31^

## AUTHOR CONTRIBUTIONS

Mohamed K. Zabady collected the clinical cases, recorded the history, performed the examination, radiography, oesophagotomy, wrote and revised the manuscript. Turke Shawaf completed the examination with endoscope, wrote and revised the manuscript.

## CONFLICTS OF INTEREST

The authors declare that they have no conflicts of interest.

## ETHICS STATEMENT

The authors confirm that the ethical polices of the journal have been followed.

## Supporting information

Supporting Information

## Data Availability

The data support the findings of this paper are available from the corresponding author upon reasonable request. The data will be anonymised and made available on reasonable request.
