# Supplementary material for: A retrospective study of choke (oesophageal obstruction) in 64 one‐hump Dromedary camels (Camelus dromedarius) in Saudi Arabia
Source: Vet Rec Open. 2022 Dec 25;9(1):e53. doi: 10.1002/vro2.53 (PMC9791241; doi:10.1002/vro2.53)
Supplement: Supplementary file 1 — Supporting Information [file VRO2-9-e53-s001.docx]

# **A retrospective study of choke (oesophageal obstruction) in 64 one-hump Dromedary camels (*Camelus dromedarius*) in Saudia Arabia**

**Supporting Information**

**Figure S1** Endoscopic view showing removal of plastic foreign body with the alligator forceps.


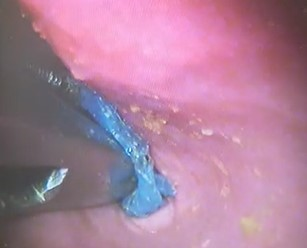


**Figure S2** (a) Piece of cloth emerging from the oesophagotomy incision in a 2-month-old male Majaheem camel calf. (b) Trichophytobezoar in the lumen of the oesophagus in a 5-year-old female Majaheem camel.

(a)


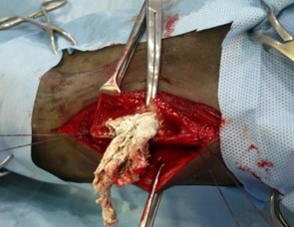

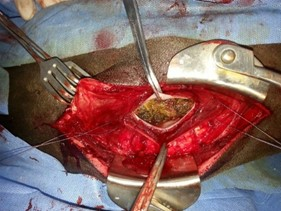


(b)

**Figure** S3 (a) Survey radiograph of the oesophagus showinga radio-opaque phytobezoar (white arrow) at the level of inter vertebral disc between fifth and sixth cervical vertebrae (black arrow) in an 8-year-old female Majaheem camel.

(b) Slightly radio-opaque piece of cloth (grey arrow) at the end of a stomach tube (white arrow) at the level of fifth cervical vertebra (black arrow) in a 2-month-old male Omani camel calf.


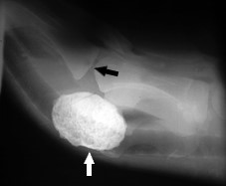

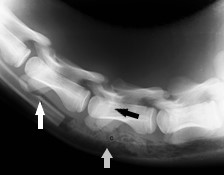


(a)

(b)

**Figure S4** (a) Plain radiograph of the oesophagus showing a radiolucent piece of cloth (grey arrow) at the end of stomach tube (white arrow) at the level of fifth cervical vertebra (black arrow) in a 2-month-old female Majaheem camel calf.

(b) Oesophagram showing the barium swallowing discontinued (white arrow) at end of the fourth cervical vertebra (black arrow) and reappeared again at the level of sixth cervical vertebra (grey arrow) with a faint shadow at fifth vertebra in a 10-day-old female Majaheem camel calf.


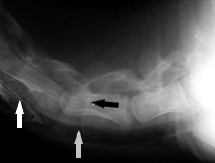

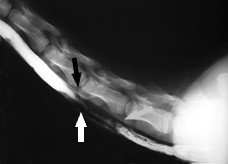


(a)

(b)

**Figure S5** The obstructive masses collected from two camels.

(a) An oval shaped phytobezoar. (b) Trichophytobezoar assumed the shape of oesophagus.


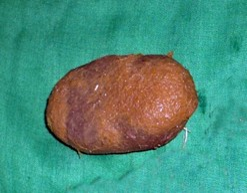

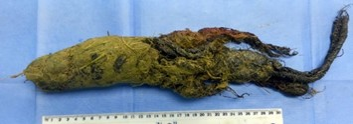


(b)

(a)
